# MiRNAs as potential biomarkers in early breast cancer detection: a systematic review

**DOI:** 10.25122/jml-2024-0322

**Published:** 2024-06

**Authors:** Ana-Maria Mihai, Laura Maria Ianculescu, Nicolae Suciu

**Affiliations:** 1 Polizu Department, Alessandrescu-Rusescu National Institute for Mother and Child Health, Bucharest, Romania; 2 Carol Davila University of Medicine and Pharmacy, Bucharest, Romania

**Keywords:** breast cancer, early detection, biomarkers, miRNAs

## Abstract

Breast cancer remains a significant global health challenge, with high incidence and mortality rates. While mammography has contributed to declining mortality, its limitations in sensitivity and specificity for early detection, particularly in distinguishing between pure atypical ductal hyperplasia (ADH), ductal carcinoma in situ (DCIS), and invasive ductal carcinoma (IDC), highlight the need for more precise tools. Even with core needle biopsy (CNB), conclusive diagnoses often require surgical excision. This underscores the urgency for non-invasive biomarkers to improve early detection and differentiation, potentially reducing invasive procedures. Recent research has shifted focus from mRNA to microRNAs (miRNAs) as promising biomarkers for breast cancer screening. These small non-coding RNAs, which exhibit abnormal expression patterns in breast cancer patients' tissue and serum/plasma, play crucial roles in early breast cancer development by modulating proto-oncogenes or tumor suppressor genes at the post-transcriptional level. Notably, miRNAs such as miR-21, miR-155, and miR-200c are key regulators of cell proliferation and apoptosis, with the potential to distinguish between normal tissue and various stages of breast lesions, including ADH, DCIS, and IDC. Additionally, miRNAs in serum and plasma offer a non-invasive method to differentiate breast cancer stages. This review aims to consolidate current knowledge on early breast lesions and explore the potential of miRNAs as biomarkers for early breast cancer detection, which could enhance risk prediction and reduce reliance on invasive diagnostic procedures.

## INTRODUCTION

With the increasing emphasis on early detection and the widespread use of mammography, there has been a significant rise in the identification of non-malignant breast lesions, such as atypical ductal hyperplasia (ADH), ductal carcinoma in situ (DCIS), atypical lobular hyperplasia (ALH), and lobular carcinoma in situ (LCIS). These lesions are increasingly recognized as substantial risk factors for the subsequent development of invasive breast carcinoma. The traditional linear progression model of ductal breast cancer, which is the most prevalent form of breast cancer, postulates a stepwise transition: beginning from normal breast epithelial cells to ductal hyperplasia, then to atypical ductal hyperplasia (ADH), progressing to ductal carcinoma in situ (DCIS), and ultimately leading to invasive ductal carcinoma (IDC) [1]. This linear progression model is supported by extensive genomic and immunohistochemical evidence, demonstrating unique molecular and cellular characteristics at each stage of disease development [2]. However, while this model has been instrumental in guiding clinical practice, it oversimplifies the intricate biological processes underlying breast cancer evolution. Emerging research in molecular genetics and immunohistochemistry has begun to unravel the complexity of breast cancer pathogenesis, revealing that the progression to invasive cancer is not strictly linear. Instead, it involves multiple, potentially divergent pathways that can lead to the development of invasive carcinoma. Recent advances have identified several alternative pathways that contribute to breast cancer development, challenging the notion of a single linear trajectory. For instance, molecular profiling has uncovered the role of various signaling pathways, such as the PI3K/AKT/mTOR and HER2/neu pathways, which may be activated at different stages, potentially leading to parallel routes of tumorigenesis [3,4]. Furthermore, studies have shown that epigenetic changes, including DNA methylation and histone modification, can also influence the transition from pre-invasive lesions to invasive cancer, adding another layer of complexity to the disease process [5,6]. In light of these findings, it is becoming increasingly clear that breast cancer is a heterogeneous disease with multiple potential origins and evolutionary paths. The characteristic genetic and cellular alterations during tumorigenesis are not simply a series of linear steps but a dynamic interplay of genetic, epigenetic, and microenvironmental factors that vary among individuals [7,8]. This nuanced understanding of breast cancer development underscores the need for more personalized approaches to risk assessment, diagnosis, and treatment, moving beyond the traditional models to consider the unique molecular and genetic landscape of each patient's disease.

The current clinical strategy for breast cancer prevention emphasizes an attempt to diagnose at the earliest possible time, as it dramatically improves the likelihood of successful treatment and favorable patient outcomes. To enhance these efforts, it is vital to deepen our understanding of early-stage cancers and the preneoplastic changes that precede them. By focusing on these early indicators, we can refine diagnostic techniques, allowing for even earlier identification of potential malignancies. This emphasis on early detection not only increases the chances of curing the disease but also offers the potential to intervene before cancer fully develops, thereby significantly reducing its impact. As research continues to unravel the complexities of these early processes, the future of breast cancer therapy may increasingly shift toward prevention and minimally invasive treatments, further improving patient prognosis and quality of life.

Mammography remains a cornerstone in breast cancer screening, primarily due to its proven ability to detect tumors at an early stage when they are more amenable to treatment, which significantly contributes to the reduction of breast cancer mortality rates. This early detection advantage has solidified mammography's role in routine clinical practice [9]. However, the effectiveness of mammography is not without challenges. One major limitation is its reduced sensitivity in women with dense breast tissue, a condition that can obscure tumors and lead to false-negative results, potentially delaying diagnosis and treatment. Additionally, the risk of overdiagnosis is a concern, as mammography may identify slow-growing or non-life-threatening cancers that result in unnecessary interventions and psychological distress for patients [10]. While the radiation exposure from mammography is relatively low, the cumulative effect of repeated screenings over a lifetime cannot be entirely dismissed, especially considering the balance between benefits and risks in different patient populations [11].

An emerging strategy to enhance breast cancer risk prediction and facilitate early detection is the analysis of molecular signatures present in normal tissue before any clinical signs of the disease manifest. Over the past decade, the focus of research has shifted from mRNA biomarkers to microRNAs (miRNAs) as promising tools for breast cancer screening. This shift is due to the discovery that miRNAs are consistently dysregulated in both tissue and serum/plasma samples from breast cancer patients. While the precise role of miRNAs in breast cancer initiation remains to be fully understood, accumulating evidence indicates that miRNAs play a crucial role in the early stages of breast cancer development and progression. They achieve this by modulating the expression of target proto-oncogenes and tumor suppressor genes (TSGs) at the post-transcriptional level [1]. These findings highlight the potential of miRNAs not only as biomarkers for early detection but also as therapeutic targets to intercept cancer development at its earliest stages.

Incorporating miRNA profiling into breast cancer screening protocols could provide a more sensitive and specific method for identifying women at high risk of developing the disease. By detecting aberrant miRNA expression patterns in normal tissue, we may be able to predict breast cancer risk long before traditional diagnostic methods can identify abnormalities, thus opening new avenues for preventive interventions and personalized treatment strategies.

### miRNA as the new biological/molecular marker

Although breast cancer research using genetic markers has shown ADH to be a definite genetic precursor to DCIS and IDC, distinguishing between different stages of breast lesions, such as ADH, DCIS, and IDC, remains a significant challenge. While genetic studies have established that atypical ductal hyperplasia (ADH) serves as a clear genetic precursor to ductal carcinoma in situ and invasive ductal carcinoma, the subtle and overlapping characteristics of these pathological phases complicate accurate differentiation. This diagnostic ambiguity highlights the need for more precise biomarkers that can offer better clarity in identifying and categorizing these lesions. In recent years, the exploration of microRNA (miRNA) expression profiles has shown great promise in bridging this gap. Numerous studies have demonstrated that miRNA dysregulation is present and varies distinctly between normal breast tissue, ADH, DCIS, and IDC, suggesting a potential role for miRNAs as reliable biomarkers in differentiating these stages. The ability to measure miRNA expression in various biological samples, including tissue, plasma, and serum, further enhances their utility in both diagnostic and prognostic settings. This flexibility allows for less invasive sampling methods, making miRNAs particularly appealing for routine clinical use. What makes miRNAs especially compelling is their functional role in regulating critical genes involved in tumorigenesis. By modulating the expression of oncogenes and tumor suppressor genes (TSGs), miRNAs can promote or inhibit cancer development depending on whether they are upregulated or downregulated. This dual role underscores their potential as therapeutic targets as well as diagnostic tools. For instance, specific miRNAs that are consistently downregulated in ADH but upregulated in IDC could serve as early warning signals for progression, guiding more personalized and timely interventions [12].

MicroRNAs are small non-coding RNA molecules, typically 18-24 nucleotides long, that play critical roles in gene regulation by binding to complementary sequences on target messenger RNAs (mRNAs), leading to their degradation or translational repression. Since their discovery, miRNAs have been extensively studied for their involvement in various biological processes, including cell differentiation, proliferation, and apoptosis. Importantly, miRNAs have emerged as key players in cancer biology, where their dysregulated expression has been linked to tumor initiation, progression, and metastasis. MicroRNAs frequently exert their regulatory effects by binding to messenger RNAs within the 3′ untranslated region (UTR) of target genes, thus influencing gene expression post-transcriptionally [13]. This interaction is pivotal in regulating a range of biological processes essential for maintaining normal cellular functions. In the context of breast cancer, specific miRNAs, such as miRNA-200c, play critical roles in modulating key processes like angiogenesis, apoptosis, cellular proliferation, and adhesion [14]. When the expression of these miRNAs is disrupted, it can lead to a cascade of cellular malfunctions, including alterations in the cell cycle and unchecked tumor growth [15].

Recent studies have expanded our understanding of specific miRNAs involved in breast cancer. For instance, miR-21 has been consistently upregulated in breast cancer patients and is known to target tumor suppressor genes, contributing to tumorigenesis [16]. Additionally, miR-155 has been implicated in regulating immune response and inflammation, processes closely linked to cancer progression [17]. The dysregulation of miRNAs like miR-200c and miR-205 has also been associated with epithelial-to-mesenchymal transition (EMT), a critical process in cancer metastasis [18].

Research conducted by Sochor *et al*., Cuk *et al*., and Li *et al*. has revealed significant variations in miRNA expression profiles between different stages of breast lesions. Specifically, their studies demonstrated that miR-155, miR-19a, miR-181b, and miR-24—miRNAs known to inhibit tumor suppressor genes (TSGs)—are markedly elevated in cases of ductal carcinoma in situ and invasive ductal carcinoma compared to normal tissue and atypical ductal hyperplasia in serum samples. Conversely, miR-571, miR-206, miR-193a-3p, miR-526b, and miR-519a, which typically suppress oncogenes, were found to be significantly reduced in ADH, DCIS, and IDC compared to normal tissue in plasma samples [15,19–23]. These findings underscore the potential of miRNA levels in serum and plasma as valuable biomarkers for breast cancer. These miRNAs could aid in the diagnostic process, but they also hold promise for distinguishing between different stages of breast cancer, such as ADH, DCIS, and IDC, particularly when used in conjunction with tissue biopsies for confirmation.

From a personal perspective, the ability to leverage miRNA profiles in blood samples represents a transformative step in breast cancer diagnostics. This approach could significantly enhance early detection and provide a more nuanced classification of breast lesions, which is crucial for tailoring treatment strategies. The integration of miRNA analysis into clinical practice could ultimately lead to more precise and individualized patient care, offering new opportunities for improving outcomes and personalizing therapeutic interventions.

### MicroRNA detection and genome-wide approaches

Several techniques have been developed and refined to detect miRNAs in breast cancer tissues, serum, and plasma, each with advantages and limitations. Many hybridization-based miRNA detection platforms are being described.

Though low throughput, the most standardized method for miRNA analysis is Northern blotting, a traditional method for miRNA detection that involves the separation of RNA by gel electrophoresis, followed by hybridization with labeled probes [24]. It has high specificity and allows for the detection of miRNA size variants, but it is a labor-intensive method with low sensitivity and requires a large amount of RNA [25].

Advanced miRNA detection techniques include oligonucleotide microarrays and real-time reverse transcription-polymerase chain reaction (RT-PCR). MiRNA microarrays are particularly powerful, allowing for the simultaneous analysis of hundreds of miRNAs across numerous samples, providing a comprehensive overview of miRNA profiles on a genome-wide scale. These arrays are invaluable for examining the global miRNA landscape and identifying differential expression patterns. On the other hand, RT-PCR offers high sensitivity and specificity for quantifying specific miRNAs, enabling precise measurement of their expression levels. By integrating miRNA data obtained from these high-throughput methods with corresponding mRNA expression profiles, researchers can uncover novel interactions between miRNAs and their target genes. This combined approach is instrumental in elucidating the complex regulatory networks that miRNAs are involved in and can potentially lead to new insights into gene regulation and disease mechanisms [26]. We mention limitations: lower sensitivity compared to qRT-PCR, potential cross-hybridization issues, and requirement of extensive data normalization [27]. Quantitative real-time PCR (qRT-PCR) is one of the most commonly used techniques for miRNA detection due to its high sensitivity, specificity, and ability to quantify miRNA expression levels. The limitations of this technique consist in the fact that it requires prior knowledge of miRNA sequences and can only analyze a limited number of miRNAs simultaneously [28].

Next-generation sequencing (NGS) provides a comprehensive and unbiased approach to miRNA profiling. It allows for sequencing all miRNAs in a sample, including novel and rare miRNAs. It shows high sensitivity, comprehensive coverage, and the ability to detect novel miRNAs. At the same time, it has a few limitations, such as high cost, requiring bioinformatics expertise, and complex data analysis [29].

Other detection techniques include bead-based flow cytometry [30] and in situ hybridization [31]. The platforms mentioned above are limited by their restriction to known miRNA structures. Bead-based flow cytometry involves using fluorescently labeled beads that bind specifically to miRNAs in a sample, allowing for their detection and quantification using flow cytometry. It shows multiplexing capability, quantitative analysis, and high throughput, but it requires specialized equipment and can be more expensive than other techniques [32]. In situ hybridization (ISH) is used to detect and localize miRNAs within tissue sections. This technique uses labeled probes that hybridize to specific miRNAs, allowing for visualization under a microscope. It provides spatial information about miRNA expression within tissues being capable to identify the specific location of microRNA expression in tissue [33].

Each of these miRNA detection methods offers unique advantages and is chosen based on the specific requirements of the study, such as the need for high sensitivity, quantification, or high-throughput analysis. As research in breast cancer progresses, the integration of these techniques will continue to enhance our understanding of miRNA roles in cancer and their potential as biomarkers.

### MicroRNAs as diagnostic, predictive, and prognostic biomarkers

The ideal biomarker in oncology should possess several key attributes: it should be easily accessible (e.g., detectable in blood or other body fluids), sensitive enough to detect even small tumors, and specific, meaning it should not be present in healthy individuals. Due to their high tissue specificity, remarkable stability in body fluids, and consistent aberrant expression across different tumor types, miRNAs are increasingly recognized as promising biomarkers for cancer diagnosis, prognosis, and therapy prediction [20]. Several studies have highlighted the diagnostic potential of miRNAs, particularly miR-21 and miR-155, which are often upregulated in various cancers, including breast, lung, and colorectal cancers. These miRNAs can differentiate cancer patients from healthy controls with high sensitivity and specificity. For instance, Khalighfard *et al*. [34] identified a panel of miRNAs, including miR-21 and miR-155, that showed elevated plasma levels in non-metastatic breast cancer patients before surgery, chemotherapy, and radiotherapy and low levels after. miR-125b has been studied for its role in predicting response to chemotherapy in breast cancer. Wang *et al*. found an association between miR-125b expression and chemoresistance, indicating an oncogenic role for this miR [35]. miR-21 is not only a diagnostic marker but also has prognostic value. Its elevated expression is correlated with poor overall survival and increased risk of recurrence in breast cancer patients. Similarly, miR-10b, which is involved in cell migration and invasion, has been linked to metastasis and poor prognosis in breast cancer [36]. miRNAs hold significant promise as biomarkers in oncology due to their tissue specificity, stability in body fluids, and dysregulated expression in cancers. They can serve as diagnostic tools for early detection, predictive markers for treatment response, and prognostic indicators for disease outcomes. Continued research and validation in clinical settings are essential to fully realize their potential in personalized cancer care.

## MATERIAL AND METHODS

For this systematic review, we conducted a comprehensive search of the PubMed database, covering publications from 2001 to the present. Our search strategy was designed to capture a broad range of relevant literature on the role of miRNAs in breast cancer. We utilized specific keywords, including 'micro-RNA' or 'miR*' in combination with 'breast cancer' and refined our search further with terms such as 'diagnostic', 'prognostic', and 'predictive', based on the focus of each review chapter. This approach allowed us to gather a wide array of studies, particularly emphasizing those with recent and robust findings validated by independent research teams.

Our selection criteria were stringent to ensure the inclusion of high-quality studies. We specifically sought articles that explored the role of miRNAs in breast cancer, using both tissue and blood samples from patients. Studies were eligible if they provided insights into how these miRNAs relate to breast cancer biology, reflecting their potential as biomarkers or therapeutic targets.

Conversely, we excluded articles that did not meet our criteria, including retracted papers, those lacking essential information about breast cancer biology, or studies focused on other cancer types. This rigorous process was critical for ensuring our review remains relevant and grounded in reliable, impactful research.

The importance of our paper lies in its ability to synthesize the latest findings on miRNAs and their role in breast cancer detection and management. We aim to highlight the most promising miRNA biomarkers and their potential clinical applications by focusing on well-validated studies and employing a meticulous selection strategy. Our approach contributes to a clearer understanding of miRNA involvement in breast cancer and offers a pathway toward more effective diagnostic and therapeutic strategies. This review represents a crucial step toward integrating miRNA research into practical clinical tools, ultimately enhancing early detection and personalized treatment for breast cancer patients.

## RESULTS

This review will delve into the primary diagnostic miRNA signatures, particularly emphasizing those validated across multiple studies or verified in diverse cohorts. This approach is crucial because it provides a more robust assessment of the potential diagnostic value of miRNAs. For instance, recent research highlighted a distinctive panel of nine circulating miRNAs that successfully differentiated early-stage breast cancer from healthy controls, underscoring the potential of miRNA profiles in early detection [37]. A meta-analysis focusing on miR-155 has demonstrated its remarkable sensitivity and specificity, reinforcing its role as a promising diagnostic biomarker [38]. Additionally, Chan and colleagues made significant strides by pinpointing four miRNAs in serum samples that emerged as pivotal diagnostic markers when comparing patients with breast cancer to healthy volunteers. This finding is particularly exciting because it suggests that a relatively small number of miRNAs could be used to identify breast cancer with high accuracy [39]. Similarly, Cuk and collaborators discovered another set of four upregulated miRNAs in the plasma of patients with breast cancer, which were effective in detecting cancers at stage I or II. These miRNAs hold great promise for enhancing early breast cancer detection and potentially improving patient outcomes through earlier intervention [40]. Moreover, another study identified three upregulated miRNAs and one downregulated miRNA in patients with breast cancer compared to normal controls. This kind of differential expression highlights the nuanced role miRNAs play in breast cancer pathology and their potential for distinguishing between cancerous and non-cancerous conditions [41]. Furthermore, a prospective study identified three significantly overexpressed serum miRNAs in women who eventually developed breast cancer compared to those who remained cancer-free. This finding suggests that these miRNAs could serve as diagnostic tools and predictors of breast cancer risk, offering valuable insights into personalized risk assessment strategies [42,43].

Khalighfard *et al*. investigated the expression levels of specific circulating microRNAs (miRs) in non-metastatic breast cancer patients before and after surgery, chemotherapy, and radiotherapy. The results show that the oncomiRs (miR-21, miR-10b, and miR-155) were significantly upregulated, while the tumor suppressor miR Let-7a was downregulated in breast cancer patients compared to healthy controls. Following treatment, oncomiRs were downregulated, and Let-7a levels increased, suggesting that monitoring these miRs could serve as valuable biomarkers for assessing treatment response and disease progression [34].

In the Lowery *et al*. study, miR-21, miR-145, and miR-155 were identified as significantly dysregulated in breast cancer, but miR-21, in particular, was upregulated in early-stage breast cancer, suggesting its utility as a strong candidate for early breast cancer biomarker development, due to its consistent upregulation in early-stage disease [43]. Another similar study analyzed plasma samples from patients with breast cancer and healthy controls, identifying miR-195 and let-7a as significantly overexpressed in breast cancer. These miRNAs showed potential as early biomarkers with high sensitivity and specificity [40].

Iorio *et al*. explored miRNA expression profiles in human breast cancer and their potential as diagnostic tools. The study identified several miRNAs, including miR-10b, miR-125b, and miR-145, significantly downregulated in breast cancer tissues compared to normal tissues, suggesting its potential as an early diagnostic marker [44].

Zheng *et al*. analyzed miRNA expression in serum samples from patients with early breast cancer and healthy controls. They identified miR-16 and miR-145 as significantly downregulated in early breast cancer. These miRNAs showed promise as biomarkers for early detection, particularly in distinguishing between cancerous and non-cancerous states [45]. Heneghan *et al*. found that circulating levels of miR-195 and miR-21 were significantly higher in patients with breast cancer compared to healthy controls. Importantly, these miRNAs could differentiate between early-stage breast cancer and benign breast conditions [46].

These findings collectively underscore the transformative potential of miRNAs as diagnostic biomarkers in breast cancer. The ability to detect and classify breast cancer at an early stage using circulating miRNAs could revolutionize current screening practices and significantly improve patient outcomes. However, while the results are promising, it is essential to continue validating these biomarkers across larger and more diverse populations to ensure their reliability and clinical utility. Our paper aims to contribute to this growing body of evidence by summarizing and analyzing the current state of miRNA research in breast cancer, ultimately guiding future efforts in this field.

## DISCUSSION

MicroRNAs have emerged as promising biomarkers for the early detection of breast cancer, offering potential as non-invasive diagnostic tools. This review examines key studies that have identified specific miRNA signatures associated with early-stage breast cancer, particularly focusing on those validated by multiple studies or across different patient cohorts. Notably, a signature of nine circulating miRNAs has shown high accuracy in distinguishing early-stage breast cancer from healthy controls. Meta-analyses have highlighted the diagnostic accuracy of miR-155, while other studies have identified miR-21, miR-145, and miR-195 as consistently dysregulated in patients with breast cancer. These miRNAs demonstrate significant potential for early detection, with miR-21 frequently noted for its ability to differentiate early-stage breast cancer from benign breast conditions. Additionally, studies have shown that miR-16, miR-10b, and let-7a, among others, can be effective in distinguishing cancerous from non-cancerous states, further supporting their role as early diagnostic markers [34,43,44,45,46].

While individual miRNAs like miR-21 and miR-155 show strong diagnostic potential, combining multiple miRNAs into a signature or panel could improve diagnostic accuracy. For instance, the combination of miR-16, miR-145, and miR-195 could potentially offer a broader detection spectrum, capturing a more comprehensive range of early-stage breast cancer cases and reducing false positives associated with benign breast conditions [43,45,46].

Despite promising results, there are significant challenges in translating these findings into clinical practice. Variability in miRNA expression due to factors such as patient demographics, tumor heterogeneity, and differences in sample collection methods can affect the reliability of these biomarkers. Standardizing protocols and conducting large-scale validation studies across diverse populations will be crucial for successfully integrating miRNAs into clinical diagnostics.

Moving forward, research should focus on developing multiplex assays that can simultaneously detect multiple miRNAs in a single test. This could enhance the sensitivity and specificity of breast cancer diagnostics. Additionally, exploring the role of circulating miRNAs in different breast cancer subtypes could provide insights into subtype-specific biomarkers, further refining early detection strategies.

## CONCLUSION

In recent years, research has increasingly focused on the regulatory role of miRNAs, a class of small non-coding RNAs, in various cancers. These molecules are now understood to contribute to the heterogeneity and pathology of different cancers through their influence on gene regulation and carcinogenic pathways. Given their association with cancer development and their potential for diagnostic and therapeutic use, miRNAs are being actively studied for their impact on disease progression.

The expanding body of research on miRNAs, particularly in the context of early breast cancer detection, underscores their potential as clinical diagnostic tools. Several miRNAs, including miR-21, miR-145, miR-155, and miR-195, have been repeatedly validated across multiple studies, confirming their accuracy in identifying early-stage breast cancer. These consistent findings suggest that miRNAs could play a crucial role in non-invasive diagnostic strategies, offering earlier and more precise detection than current methods.

However, despite the promising results, challenges remain in firmly establishing miRNAs as reliable biomarkers. Further validation in independent cohorts and through additional studies is critical. Additionally, multivariate testing is essential, as individual miRNAs may lose significance when analyzed in the context of multiple variables. Differences in cancer subtype distribution or cells of origin may also contribute to inconsistencies in results. Therefore, more comprehensive experimental evidence is needed to strengthen the utility of miRNAs in clinical decision-making.
